# Bloating, Diarrhoea and Maldigestion in Patients with Metabolic Syndrome: Are Fatty Pancreas and Pancreatic Exocrine Insufficiency the Missing Pieces of the Puzzle?

**DOI:** 10.3390/jcm11195720

**Published:** 2022-09-27

**Authors:** Amir Mari, Wisam Sbeit, Tawfik Khoury

**Affiliations:** 1 Gastroenterology Department, Nazareth Hospital, Azrieli Faculty of Medicine, Bar Ilan University, Ramat Gan 5290002, Israel; 2 Gastroenterology Department, Galilee Medical Center, Azrieli Faculty of Medicine, Bar Ilan University, Ramat Gan 5290002, Israel

Pancreatic exocrine insufficiency (PEI) is a disorder causing symptoms such as maldigestion, malnutrition, diarrhoea, bloating, vitamin deficiency and weight loss [1]. PEI is caused by inability of the enzymatic secretory function of the pancreas, due to parenchymal disease and/or due to obstruction of the main pancreatic duct, to lead the enzymes and bicarbonates to the gut [2,3]. The pancreatic diseases that may progress to PEI include chronic pancreatitis, cystic fibrosis, pancreatic cystic lesions, diabetes mellitus, pancreatic cancer and severe/necrotizing pancreatitis [4]. Importantly, extra-pancreatic and systemic disorders may cause PEI as well, including celiac disease, bariatric surgeries, chronic kidney disease, and inflammatory bowel diseases, as well as medication side effects [4]. Fatty pancreas (FP) is an evolving medical entity specified by parenchymal fat deposition within the pancreatic tissue [5] with an increasing prevalence, probably due to the widespread use of imaging studies as well as the globally increasing prevalence of metabolic syndrome [5,6]. The clinical relevance of FP is still not entirely known; however, some studies have linked FP to metabolic syndrome, diabetes mellitus, cardiovascular disease, fatty liver, and to pancreatic cancer [7,8,9,10,11,12,13,14,15,16]. Moreover, the possible association between fatty pancreas and PEI is very interesting and has attracted some focus in recent years. This link could represent an important explanation for some ‘idiopathic’ cases of PEI and allow better patient diagnosis and treatment, particularly for patients with mild symptoms of abdominal pain, diarrhoea and maldigestion that could be misdiagnosed as irritable bowel disease (IBS)-predominant diarrhoea, whereas they have some component of PEI [17]. The literature on the possible causative association between FP and PEI is very limited and mainly relies on observational/retrospective data, whereas laboratory/clinical interventional well-designed trials are lacking. Some studies have proposed a possible correlation between FP and PEI [18,19,20,21,22]. A German population-based study that included 1458 healthy volunteers that had undergone MRI, and who had all performed the faecal elastase test, aimed to assess the connection between fatty pancreas infiltration and PEI as revealed by the low faecal elastase (<200 microgram/gr stool). The key result of this study is the contrary connection between the quantity of fat accumulation within pancreatic parenchyma and the faecal elastase level, suggesting a promising causative association between FP and PEI [23]. On the other hand, some reports have refuted this possible link. Nonetheless, when cautiously noting the published data, heterogeneous and observational, weak evidence studies are mainly available. To conclude, the possible link between FP and PEI is very interesting and should be better addressed and studied for better patient management and care. Therefore, well-designed clinical trials are eagerly merited for better exploration of the potential link between FP and PEI.
